# A novel p.F206I mutation in Cx46 associated with autosomal dominant congenital cataract

**Published:** 2012-04-18

**Authors:** Kai Jie Wang, Si Quan Zhu

**Affiliations:** Beijing Tongren Eye Center, Beijing Tongren Hospital, Capital Medical University, Beijing Ophthalmology & Visual Sciences Key Laboratory, Beijing, China

## Abstract

**Purpose:**

To identify the genetic defect in a Chinese family with bilateral congenital cataract.

**Methods:**

A three-generation family was recruited in this study. Detailed family history and clinical data were recorded. Ten candidate genes were screened for causative mutations. Direct sequencing was performed to analyze the cosegregation of the genotype with the disease phenotype.

**Results:**

Affected individuals presented embryonal nuclear opacities in the lens. Sequencing of the candidate genes showed a heterozygous c. 616T>A variation in the connexin 46 (*Cx46*) gene, which resulted in the replacement of a highly conserved phenylalanine by isoleucine at codon 206 (p. F206I). This mutation co-segregated with all affected individuals and was not observed in unaffected family members or ethnically matched controls.

**Conclusions:**

We report a novel mutation (p.F206I) in the fourth transmembrane domain of connexin 46. These findings thus expand the mutation spectrum of *Cx46* in association with congenital cataract.

## Introduction

Congenital cataract is defined as any opacity of the lens, which is present from birth and is responsible for approximately one-tenth of worldwide childhood blindness [1,2]. About one third of isolated congenital cataracts are genetically determined. Autosomal dominant congenital cataract (ADCC) is the most common mode of inheritance, although autosomal recessive and X-linked inheritance are also known to exist [3].

To date, more than 34 loci and 18 genes on different chromosomes have been associated with isolated ADCC [2,4]. Of the cataract mutations reported to date, about half have mutations in crystallins, a quarter have mutations in connexins, and the remainder is evenly divided between intrinsic membrane proteins, intermediate filament proteins, transcription factors and other genes [5]. Hansen et al. [6] detected crystallin and connexin mutations in 35.7% (10/28) and 21.4% (6/28) Danish families, respectively. Sun et al. [7] detected mutations in 40% (10/25) Chinese families by analyzing the 12 genes encoding crystallins and connexins. Our previous study also identified β-crystalline mutations in 15% (3/20) Chinese families with congenital nuclear cataract [8]. Therefore, the crystalline and connexin genes appear to be the most common genes associated with congenital cataract. It is appropriate to consider these genes as the top list of candidate genes for screening studies in congenital cataracts.

In the present study, we screened the 8 crystalline and 2 connexin genes using the same strategy as described previously [8]. A novel missense mutation in connexin 46 (*Cx46*) that co-segregated with the disease phenotype was identified to be responsible for ADCC.

## Methods

### Clinical evaluation and DNA specimens

A three-generation family from Hebei province, China with autosomal dominant nuclear cataract was identified. Both affected and unaffected individuals underwent detailed ophthalmic examinations including visual acuity, slit lamp examination, ultrasonography, fundus examination, and intraocular pressure measurement. The phenotypes were documented by slit lamp photography. A total of 110 unrelated ethnically matched controls with no family history of congenital cataracts were also recruited. This study was conducted in accordance with the tenets of the Declaration of Helsinki and approved by the ethics committees for medical research at Capital Medical University, Beijing, China. After informed consents, peripheral venous blood of all participants was collected and DNA was extracted using a QIAamp DNA kit (Qiagen, Valencia, CA) according to the manufacturer’s instructions. A 200 µl aliquot of blood sample was incubated with QIAGEN protease and buffer AL at 56 °C for10 min. The lysate was applied to a QIAamp spin column, and washed twice with buffer AW and finally eluted with 200 µl of Buffer AE.

### Mutation analysis

Mutation screening was performed in 10 candidate genes: αA-crystallin (*CRYAA*; GenBank NM_000394), αB-crystallin (*CRYAB* ;GenBank NM_001885), βA1-crystallin (*CRYBA1*; GenBank NM_005208), βB1-crystallin (*CRYBB1*; GenBank NM_001887), βB2-crystallin (*CRYBB2*; GenBank NM_000496), γC-crystallin (*CRYGC*; GenBank NM_020989), γD-crystallin (*CRYGD*; GenBank NM_006891), γS-crystallin (*CRYGS*; GenBank NM_017541), Connexin 46 (*Cx46*; GenBank NM_021954), and Connexin 50 (*Cx50*; GenBank NM_005267). All coding exons and splice sites of the candidate genes were amplified by polymerase chain reactions (PCR) using previously reported primer sequences (Table 1) [8]. PCR was performed in a final volume of 25 μl containing 1× PCR buffer (Invitrogen™ Life Technology, Carlsbad, CA), 1.5 mM MgCl_2_, 0.2 mM of dNTP, 0.2 μM of each primers, 0.5 U of Platinum® Taq DNA polymerase (Invitrogen). The PCR products obtained from the proband and one unaffected member were sequenced on an ABI3730 Automated Sequencer (PE Biosystems, Foster City, CA). Direct sequencing was also used to screen the mutation identified in *Cx46* on the sample of all available family members and 110 ethnically matched controls to confirm the mutation.

**Table 1:** Primer sequences for PCR amplification

**Gene**	**Forward Primers (5′→3′)**	**Reverse Primers (5′→3′)**	**Annealing Temperature (°C)**	**Product Size (bp)**
*CRYAA-1*	AGCAGCCTTCTTCATGAGC	CAAGACCAGAGTCCATCG	62	584
*CRYAA-2*	GGCAGGTGACCGAAGCATC	GAAGGCATGGTGCAGGTG	62	550
*CRYAA-3*	GCAGCTTCTCTGGCATGG	GGGAAGCAAAGGAAGACAGA	62	511
*CRYAB-1*	AACCCCTGACATCACCATT	AAGGACTCTCCCGTCCTAG	62	352
*CRYAB-2*	CCATCCCATTCCCTTACCT	GCCTCCAAAGCTGATAGCA	60	237
*CRYAB-3*	TCTCTCTGCCTCTTTCCTC	CCTTGGAGCCCTCTAAATC	60	477
*CRYBA1–1*	GGCAGAGGGAGAGCAGAGTG	CACTAGGCAGGAGAACTGGG	60	550
*CRYBA1–2*	AGTGAGCAGCAGAGCCAGAA	GGTCAGTCACTGCCTTATGG	60	508
*CRYBA1–3*	AAGCACAGAGTCAGACTGAAGT	CCCCTGTCTGAAGGGACCTG	60	463
*CRYBA1–4*	GTACAGCTCTACTGGGATTG	ACTGATGATAAATAGCATGAACG	60	355
*CRYBA1–5*	GAATGATAGCCATAGCACTAG	TACCGATACGTATGAAATCTGA	60	597
*CRYBA1–6*	CATCTCATACCATTGTGTTGAG	CATCTCATACCATTGTGTTGAG	62	528
*CRYBB1-1*	CCCTGGCTGGGGTTGTTGA	TGCCTATCTGCCTGTCTGTTTCTC	58	620
*CRYBB1-2*	TAGCGGGGTAATGGAGGGTG	AGGATAAGAGTCTGGGGAGGTGG	58	664
*CRYBB1-3*	CCTGCACTGCTGGCTTTTATTTA	TCTCCAGAGCCCAGAACCATG	60	475
*CRYBB1-4*	CCAACTCCAAGGAAACAGGCATA	CCTCCCTACCCACCATCATCTC	60	491
*CRYBB1-5*	TAGACAGCAGTGGTCCCTGGAGA	AGCACTGGGAGACTGTGGAAGG	60	416
*CRYBB1-6*	CCTAGAAAAGGAAACCGAGGCC	AGCGAGGAAGTCACATCCCAGTA	60	551
*CRYBB2–1*	GTTTGGGGCCAGAGGGGAGTGGT	TGGGCTGGGGAGGGACTTTCAGT	62	349
*CRYBB2–2*	CCTTCAGCATCCTTTGGGTTCTCT	GCAGTTCTAAAAGCTTCATCAGTC	62	330
*CRYBB2–3*	GTAGCCAGGATTCTGCCATAGGAA	GTGCCCTCTGGAGCATTTCATAGT	62	360
*CRYBB2–4*	GGCCCCCTCACCCATACTCA	CTTCCCTCCTGCCTCAACCTAATC	62	230
*CRYBB2–5*	CTTACCCTTGGGAAGTGGCAATGG	TCAAAGACCCACAGCAGACAAGTT	62	600
*CRYGC-1*	TGCATAAAATCCCCTTACCG	CCTCCCTGTAACCCACATTG	62	514
*CRYGC-2*	TGGTTGGACAAATTCTGGAAG	CCCACCCCATTCACTTCTTA	60	430
*CRYGD-1*	CAGCAGCCCTCCTGCTAT	GGGTCCTGACTTGAGGATGT	60	550
*CRYGD-2*	GCTTTTCTTCTCTTTTTATTTCTGG	AAGAAAGACACAAGCAAATCAGT	62	308
*CRYGS-1*	GAAACCATCAATAGCGTCTAAATG	TGAAAAGCGGGTAGGCTAAA	60	229
*CRYGS-2*	AATTAAGCCACCCAGCTCCT	GGGAGTACACAGTCCCCAGA	60	319
*CRYGS-3*	GACCTGCTGGTGATTTCCAT	CACTGTGGCGAGCACTGTAT	60	491
*Cx46–1*	CGGTGTTCATGAGCATTTTC	CTCTTCAGCTGCTCCTCCTC	60	450
*Cx46–2*	GAGGAGGAGCAGCTGAAGAG	AGCGGTGTGCGCATAGTAG	60	450
*Cx46–3*	TCGGGTTCCCACCCTACTAT	TATCTGCTGGTGGGAAGTGC	62	300
*Cx50–1*	CCGCGTTAGCAAAAACAGAT	CCTCCATGCGGACGTAGT	62	420
*Cx50–2*	GCAGATCATCTTCGTCTCCA	GGCCACAGACAACATGAACA	62	330
*Cx50–3*	CCACGGAGAAAACCATCTTC	GAGCGTAGGAAGGCAGTGTC	62	350
*Cx50–4*	TCGAGGAGAAGATCAGCACA	GGCTGCTGGCTTTGCTTAG	62	500

### Bioinformatics analysis

The CLC Free Workbench 4.5.1 software (CLC bio, Aarhus, Denmark) was used to align the protein sequences from several different species. The possible impact of an amino acid substitution on the structure and function of the protein was predicted by Polyphen-2.

## Results

### Clinical findings

**Figure 1:**
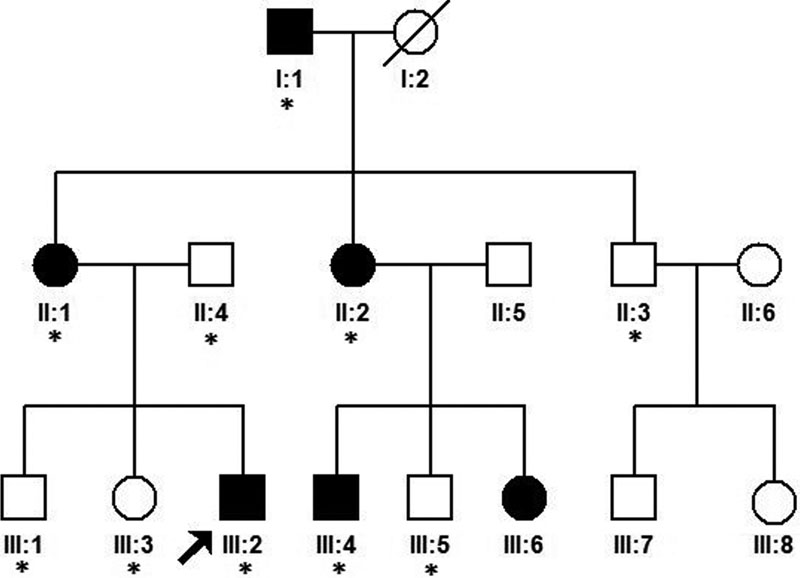
Pedigree of a cataract family. Pedigree of a three-generation family with congenital cataract. The black arrow indicates the proband. The asterisk indicates family members who attend this study.

**Figure 2:**
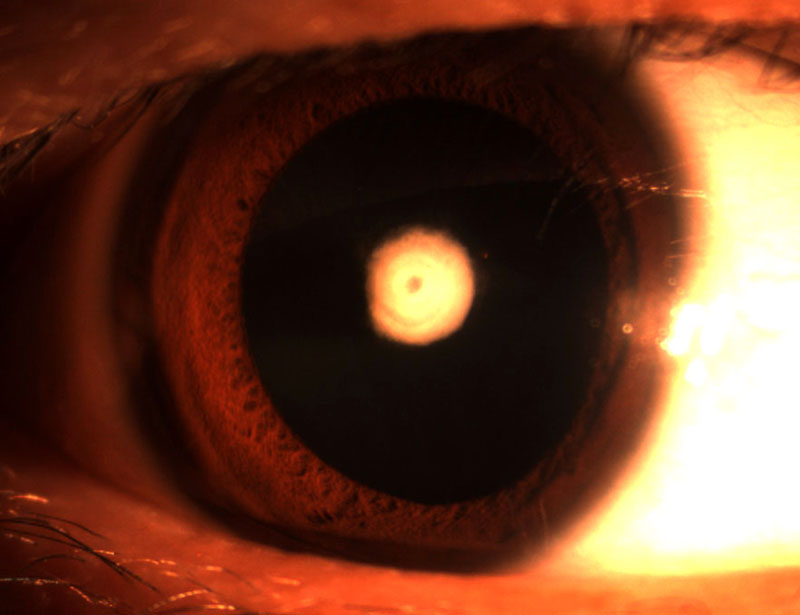
Slit lamp photographs of the proband. The photograph of the proband (III: 2) shows nuclear opacities of the lens involving embryonal nucleus.

We identified a three-generation Chinese family with autosomal dominant nuclear cataract (Figure 1). In total 10 family members (5 affected and 5 unaffected) participated in the study. The proband (III: 2) was 7 years old and dignosed with bilateral nuclear cataract at the age of 4 years. The dense nuclear opacities were located in the embryonal nucleus (Figure 2). According to the medical records, the other affected individuals were diagnosed with bilateral nuclear cataract and had cataract extraction performed. There were no other ocular or systemic abnormalities in this family.

### Mutation analysis

**Figure 3:**
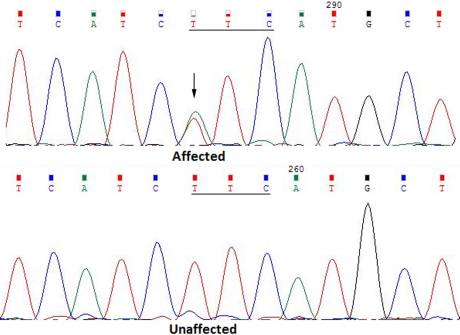
DNA sequence chromatograms of an unaffected member and an affected member in the family (Forward strand; individual III: 2 and III: 3, respectively). A single transversion is observed at position 616 (T>A) as a T /A double peak (indicated by an arrow).

Direct sequencing of the coding regions of the candidate genes in the affected individuals identified a novel heterozygous c. 616T>A variation in *Cx46* (Figure 3), which resulted in a substitution of phenylalanine to isoleucine at codon 206 (p. F206I). The substitution was not found in any of the unaffected family members or in the 110 unrelated controls.

### Bioinformatics analysis

**Figure 4:**
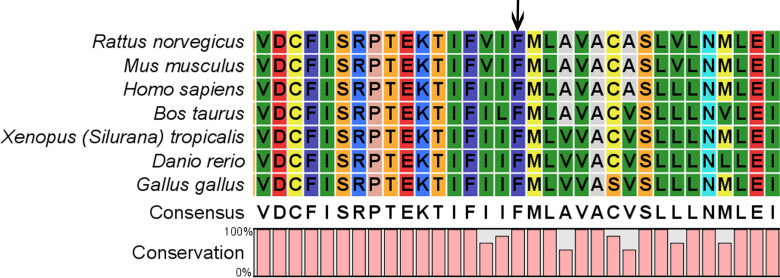
A multiple-sequence alignment in connexin 46 from different species. The alignment data indicates that the Phe at position 206 is highly conserved in different species (indicated by an arrow).

The Phe at position 206 of human connexin 46 was located within a phylogenetically conserved region by multiple-sequence alignment (Figure 4). The p. F206I was predicted to be “probably damaging” by Polyphen-2 analysis with a score of 1.000.

## Discussion

In this study, we identified a novel mutation (c. 616T>A) in *Cx46* associated with congenital cataract in a Chinese family. This variation seemed to be disease causative as it segregated completely with the disease phenotype and was absent in unaffected individuals in this family and in the 110 unrelated ethnically matched controls.

*Cx46* consists of a single exon encoding a 435 amino acid protein in humans which is essential for maintaining lens transparency. Connexins are a family of structurally-related transmembrane proteins that assemble to form gap junctions, which are used to transport metabolites, ions and water in the lens [9]. All connexins have four transmembrane domains (M1, M2, M3, and M4), two extracellular loops (E1 and E2), and three intracellular regions (the NH_2_-terminus, a cytoplasmic loop and COOH-terminus) [10]. Six connexin protein subunits oligomerize to form one hemichannel. The c. 616T>A substitution observed in the present study results in the replacement of phenylalanine to isoleucine at codon 206 (p. F206I), localized in the fourth transmembrane domain (M4) of the connexin 46. To our knowledge, this is the first identified mutation that lies in the M4 domain of the connexin 46 associated with congenital cataract.

The F206 residue of connexin 46 is phylogenetically conserved in different species, and Polyphen-2 showed that the p. F206I mutation in connexin 46 is likely to be damaging. These data indicate that the phenylalanine is likely to be functionally important and that the mutation may have a detrimental physiologic effect. The transmembrane domains of the connexins are proposed to participate in the oligomerization into hemichannels and are also important for the correct transport of the protein into the plasma membrane [11]. It has been showed that residues in the first transmembrane domain of connexin 46 are essential for the formation of the pore lining and channel permeability [12]. In addition, the p. C202F mutation in *Cx26*, which lies in the fourth transmembrane domain of connexin 26, has been reported in association with isolated autosomal dominant hearing impairment, and the authors hypothesize that the mutation may impair the connexin oligomerisation [13]. Given the p. F206I mutation affects the fourth transmembrane domain, we speculate that like other dominantly transmitted mutations in connexins, the p.F206I mutation may disturb the interaction between the M4 domain of one mutant connexin 46 and the M2 domain of the neighboring connexin, thus resulting in the formation of a non-functional channel. Further functional expression studies will be required to elucidate the precise pathogenic mechanisms that link *Cx46* mutations with congenital cataract.

In the animal model study, the targeted replacement of connexin 50 (Cx50) with the connexin 46 coding region in mice demonstrates that Cx50 is required for cell growth whereas Cx46 provides nonspecific restoration of intercellular communication [14]. Mutations in *Cx46* and *Cx50* have been demonstrated to be one of the common causes for different types of congenital cataracts in humans [15]. Apart from the mutation p. F206I, at least 18 mutations in *Cx46* have been reported to be associated with congenital cataract, which have recently been summarized by Zhang et al. [16]. The phenotypes in most of the cases have been described as pulverulent cataracts, either predominantly in the nuclear or lamellar regions of the lens. The cataract phenotype in the present family differs from these as no “pulverized” dense embryonal opacities are showed in the lens. Chang et al. [17] have found that mice with heterozygous and homozygous *Cx50* mutations display different types of cataracts, such as nuclear cataracts, cortical cataracts or lens posterior rupture. Therefore, different types of cataracts may be caused by altered intercellular communication mediated by diverse gap junction channels consisting of mutant and wild-type connexin subunits in the lens [18].

In summary, we describe a novel p. F206I mutation in *Cx46* associated with nuclear cataract of Chinese origin. These findings further expand the genetic and phenotypic heterogeneity of congenital cataract.
